# (*E*)-5-Chloro-3-(2,6-dichloro­benzyl­idene)­indolin-2-one

**DOI:** 10.1107/S1600536808031930

**Published:** 2008-10-11

**Authors:** Hongming Zhang, Haribabu Ankati, Shashidhar Kumar Akubathini, Ed Biehl

**Affiliations:** a Department of Chemistry, Southern Methodist University, Dallas, TX 75275, USA

## Abstract

There are two independent mol­ecules of the title compound, C_15_H_8_Cl_3_NO, in the asymmetric unit. Both form inversion dimers via pairs of hydrazide–carbonyl N—H⋯O hydrogen bonds.

## Related literature

For background information on the pharmacological activities of 3-substituted indoline-2-ones, see: Andreani *et al.* (2006 ▶); Sun *et al.* (2003 ▶); Johnson *et al.* (2005 ▶). For related structures, see: Gayathri *et al.* (2008 ▶); Ali *et al.* (2008 ▶); De (2008 ▶).
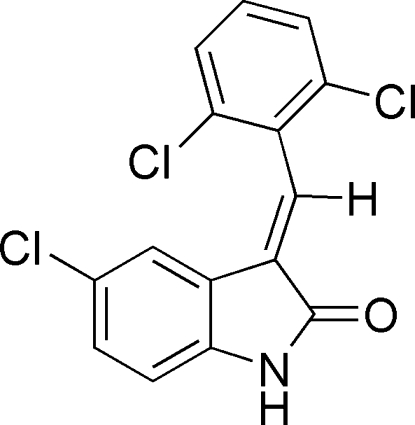


## Experimental

### 

#### Crystal data

C_15_H_8_Cl_3_NO
*M* *_r_* = 324.57
Triclinic, 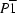

*a* = 8.0809 (6) Å
*b* = 13.4944 (11) Å
*c* = 14.3698 (16) Å
α = 63.116 (1)°
β = 82.973 (2)°
γ = 80.162 (1)°
*V* = 1375.3 (2) Å^3^
*Z* = 4
Mo *K*α radiation
μ = 0.66 mm^−1^
*T* = 296 (2) K
0.39 × 0.28 × 0.21 mm

#### Data collection

Bruker APEX CCD area-detector diffractometer
Absorption correction: multi-scan (*SADABS*; Sheldrick, 1996 ▶) *T*_min_ = 0.785, *T*_max_ = 0.874
16659 measured reflections
6438 independent reflections
5292 reflections with *I* > 2σ(*I*)
*R*_int_ = 0.028

#### Refinement

*R*[*F* ^2^ > 2σ(*F* ^2^)] = 0.060
*wR*(*F* ^2^) = 0.158
*S* = 1.08
6438 reflections
361 parameters
H-atom parameters constrained
Δρ_max_ = 0.53 e Å^−3^
Δρ_min_ = −0.39 e Å^−3^

### 

Data collection: *SMART* (Bruker, 1997 ▶); cell refinement: *SAINT* (Bruker, 1997 ▶); data reduction: *SAINT*; program(s) used to solve structure: *SHELXS97* (Sheldrick, 2008 ▶); program(s) used to refine structure: *SHELXL97* (Sheldrick, 2008 ▶); molecular graphics: *SHELXTL* (Sheldrick, 2008 ▶); software used to prepare material for publication: *SHELXTL* and *publCIF* (Westrip, 2008 ▶).

## Supplementary Material

Crystal structure: contains datablocks I, global. DOI: 10.1107/S1600536808031930/jh2068sup1.cif

Structure factors: contains datablocks I. DOI: 10.1107/S1600536808031930/jh2068Isup2.hkl

Additional supplementary materials:  crystallographic information; 3D view; checkCIF report

## Figures and Tables

**Table 1:** Hydrogen-bond geometry (Å, °)

*D*—H⋯*A*	*D*—H	H⋯*A*	*D*⋯*A*	*D*—H⋯*A*
N1—H1⋯O2^i^	0.86	2.09	2.919 (3)	162
N21—H21⋯O22^ii^	0.86	2.02	2.838 (3)	159

Symmetry codes: (i) 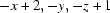; (ii) 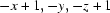.

**Fig. 1.**
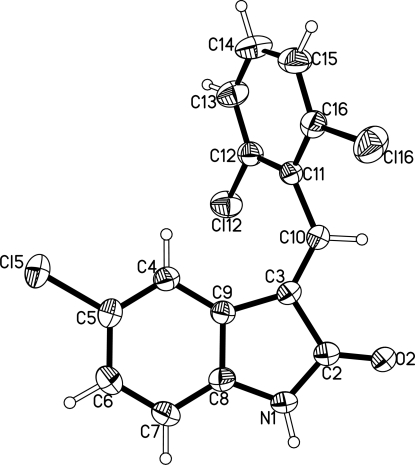
A view of one of the independent molecules with displacement ellipsoids drawn at the 40% probability level. H atoms are presented as open circles with arbitrary radii. Atoms of another independent molecule were labeled as N21 C22 O22 C23 through C35 C36 Cl36.

**Fig. 2.**
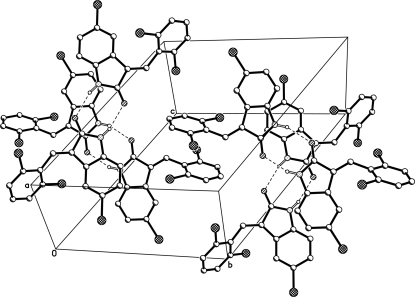
A unit cell packing view of the title compound. Dash lines indicate hydrogen bonds. For clarity, H atoms not involved in H-bonding were omitted.

## supplementary crystallographic information

### Comment

3-Substituted indoline-2-ones have well recognized pharmacological activities, including antitumor properties (Andreani *et al.*, 2006), receptor tyrosine kinase (RTK) inhibitors (Sun *et al.*, 1998) and neuroprotective agents (D'Mello *et al.*, 2005). To study their neuroprotective activity, a series of 3- and 3,5-substituted indoline-2-one derivatives have been synthesized and crystallized in our laboratory. As part of our studies on structure–activity relationships of 3-substituted indoline-2-ones and the importance of substituent at the 5-postion, the title compound was synthesized and its crystal structure was carried out. The study found the title compound adopted an *E* conformation in the structure (Fig. 1) and that converted into a mixture of *E* and *Z* isomers in DMSO-*d*_6_ solution. As expected, the substituent, O2, Cl5, and C10, lie essentially in the plane of the indole ring. The indolyl plane with that of phenyl are twisted, with the dihedral angles between the rings are 62.16 (10), 63.06 (6)°, respectively, for each independent molecule. It's similar to other indolin-2-one compounds (Gayathri *et al.*, 2008; Ali *et al.*, 2008; De, 2008) containing intermolecular hydrazide–carbonyl N—H···O hydrogen bonds. The H-bonds link two inverted molecules and a dimer is formed (Table 1).

### Experimental

The title compound was synthesized by the condensation of 2,6-dichlorobenzaldehyde (1 mmol) with 5-chloro-oxindole (1 mmol) in ethanol (10 ml) in the presence of catalytic amount of piperidine (0.1 mmol). After refluxing for 3 hrs, the reaction mixture was left to stand overnight. The resulting crude solid was filtered, washed with cold ethanol (10 ml) and dried. Orange colored single crystals of the compound suitable for X-ray structure determination were recrystallized from ethanol.

### Crystal data

**Table:**

C_15_H_8_Cl_3_NO	*Z* = 4
*M**_r_* = 324.57	*F*(000) = 656
Triclinic, *P*1	*D*_x_ = 1.568 Mg m^−^^3^
Hall symbol: -P 1	Mo *K*α radiation, λ = 0.71073 Å
*a* = 8.0809 (6) Å	Cell parameters from 9259 reflections
*b* = 13.4944 (11) Å	θ = 2.6–28.2°
*c* = 14.3698 (16) Å	µ = 0.66 mm^−^^1^
α = 63.116 (1)°	*T* = 296 K
β = 82.973 (2)°	Rod, orange
γ = 80.162 (1)°	0.39 × 0.28 × 0.21 mm
*V* = 1375.3 (2) Å^3^	

### Data collection

**Table:**

Bruker APEX CCD area-detector diffractometer	6438 independent reflections
Radiation source: fine-focus sealed tube	5292 reflections with *I* > 2σ(*I*)
graphite	*R*_int_ = 0.028
Detector resolution: 83.33 pixels mm^-1^	θ_max_ = 28.2°, θ_min_ = 1.6°
φ and ω scans	*h* = −10→10
Absorption correction: multi-scan (SADABS; Sheldrick, 1996)	*k* = −17→17
*T*_min_ = 0.785, *T*_max_ = 0.874	*l* = −18→18
16659 measured reflections	

### Refinement

**Table:**

Refinement on *F*^2^	Primary atom site location: structure-invariant direct methods
Least-squares matrix: full	Secondary atom site location: difference Fourier map
*R*[*F*^2^ > 2σ(*F*^2^)] = 0.060	Hydrogen site location: inferred from neighbouring sites
*wR*(*F*^2^) = 0.158	H-atom parameters constrained
*S* = 1.08	*w* = 1/[σ^2^(*F*_o_^2^) + (0.0786*P*)^2^ + 0.7214*P*] where *P* = (*F*_o_^2^ + 2*F*_c_^2^)/3
6438 reflections	(Δ/σ)_max_ < 0.001
361 parameters	Δρ_max_ = 0.53 e Å^−^^3^
0 restraints	Δρ_min_ = −0.39 e Å^−^^3^

### Special details

**Table:**

Experimental. All e.s.d.'s (except the e.s.d. in the dihedral angle between two l.s. planes) are estimated using the full covariance matrix. The cell e.s.d.'s are taken into account individually in the estimation of e.s.d.'s in distances, angles and torsion angles; correlations between e.s.d.'s in cell parameters are only used when they are defined by crystal symmetry. An approximate (isotropic) treatment of cell e.s.d.'s is used for estimating e.s.d.'s involving l.s. planes.
Geometry. All e.s.d.'s (except the e.s.d. in the dihedral angle between two l.s. planes) are estimated using the full covariance matrix. The cell e.s.d.'s are taken into account individually in the estimation of e.s.d.'s in distances, angles and torsion angles; correlations between e.s.d.'s in cell parameters are only used when they are defined by crystal symmetry. An approximate (isotropic) treatment of cell e.s.d.'s is used for estimating e.s.d.'s involving l.s. planes.
Refinement. Refinement of *F*^2^ against ALL reflections. The weighted *R*-factor *wR* and goodness of fit *S* are based on *F*^2^, conventional *R*-factors *R* are based on *F*, with *F* set to zero for negative *F*^2^. The threshold expression of *F*^2^ > σ(*F*^2^) is used only for calculating *R*-factors(gt) *etc*. and is not relevant to the choice of reflections for refinement. *R*-factors based on *F*^2^ are statistically about twice as large as those based on *F*, and *R*- factors based on ALL data will be even larger.

### Fractional atomic coordinates and isotropic or equivalent isotropic displacement parameters (Å^2^)

**Table:**

	*x*	*y*	*z*	*U*_iso_*/*U*_eq_	
N1	0.8654 (3)	0.07885 (18)	0.38440 (16)	0.0441 (5)	
H1	0.8834	0.0942	0.4340	0.053*	
C2	0.9186 (3)	−0.0212 (2)	0.38197 (19)	0.0419 (5)	
O2	1.0019 (3)	−0.10122 (17)	0.44698 (16)	0.0564 (5)	
C3	0.8546 (3)	−0.0123 (2)	0.28386 (18)	0.0366 (5)	
C4	0.6911 (3)	0.1609 (2)	0.13832 (19)	0.0407 (5)	
H4	0.6838	0.1275	0.0950	0.049*	
C5	0.6255 (4)	0.2711 (2)	0.1105 (2)	0.0465 (6)	
Cl5	0.53114 (14)	0.34756 (7)	−0.00817 (7)	0.0780 (3)	
C6	0.6336 (4)	0.3220 (2)	0.1745 (2)	0.0532 (7)	
H6	0.5871	0.3964	0.1540	0.064*	
C7	0.7100 (4)	0.2633 (2)	0.2680 (2)	0.0497 (6)	
H7	0.7157	0.2968	0.3114	0.060*	
C8	0.7778 (3)	0.1536 (2)	0.29584 (19)	0.0399 (5)	
C9	0.7679 (3)	0.10133 (19)	0.23239 (18)	0.0355 (5)	
C10	0.8876 (3)	−0.0984 (2)	0.26019 (19)	0.0396 (5)	
H10	0.9537	−0.1608	0.3067	0.047*	
C11	0.8348 (3)	−0.10953 (18)	0.17102 (18)	0.0358 (5)	
C12	0.6685 (3)	−0.1043 (2)	0.15124 (19)	0.0405 (5)	
Cl12	0.50744 (9)	−0.07520 (7)	0.23006 (6)	0.0552 (2)	
C13	0.6254 (4)	−0.1275 (3)	0.0740 (2)	0.0534 (7)	
H13	0.5128	−0.1237	0.0632	0.064*	
C14	0.7491 (4)	−0.1563 (3)	0.0131 (2)	0.0614 (8)	
H14	0.7202	−0.1718	−0.0393	0.074*	
C15	0.9149 (4)	−0.1623 (3)	0.0288 (2)	0.0559 (7)	
H15	0.9991	−0.1817	−0.0124	0.067*	
C16	0.9551 (3)	−0.1393 (2)	0.1065 (2)	0.0426 (5)	
Cl16	1.16558 (10)	−0.14706 (9)	0.12559 (7)	0.0693 (3)	
N21	0.3669 (3)	0.12638 (17)	0.40122 (17)	0.0442 (5)	
H21	0.3913	0.0600	0.4054	0.053*	
C22	0.4168 (4)	0.1590 (2)	0.4684 (2)	0.0441 (6)	
O22	0.5053 (3)	0.10166 (16)	0.54222 (16)	0.0602 (6)	
C23	0.3436 (3)	0.28014 (19)	0.43404 (19)	0.0411 (5)	
C24	0.1590 (4)	0.4078 (2)	0.2758 (2)	0.0457 (6)	
H24	0.1457	0.4719	0.2863	0.055*	
C25	0.0844 (4)	0.4072 (2)	0.1947 (2)	0.0522 (7)	
Cl25	−0.04299 (14)	0.52723 (8)	0.11626 (7)	0.0819 (3)	
C26	0.1041 (4)	0.3135 (3)	0.1766 (2)	0.0560 (7)	
H26	0.0546	0.3164	0.1201	0.067*	
C27	0.1976 (4)	0.2155 (2)	0.2423 (2)	0.0517 (7)	
H27	0.2107	0.1517	0.2313	0.062*	
C28	0.2706 (3)	0.2145 (2)	0.32410 (19)	0.0412 (5)	
C29	0.2540 (3)	0.3105 (2)	0.34098 (19)	0.0399 (5)	
C30	0.3666 (3)	0.3334 (2)	0.48909 (19)	0.0433 (6)	
H30	0.4207	0.2894	0.5511	0.052*	
C31	0.3183 (3)	0.4519 (2)	0.46532 (19)	0.0414 (6)	
C32	0.3765 (4)	0.5398 (2)	0.3753 (2)	0.0451 (6)	
Cl32	0.50911 (12)	0.51303 (6)	0.28279 (6)	0.0612 (2)	
C33	0.3398 (4)	0.6498 (2)	0.3583 (2)	0.0524 (7)	
H33	0.3818	0.7061	0.2979	0.063*	
C34	0.2410 (4)	0.6762 (2)	0.4306 (3)	0.0569 (8)	
H34	0.2143	0.7507	0.4185	0.068*	
C35	0.1809 (4)	0.5927 (2)	0.5213 (3)	0.0562 (7)	
H35	0.1142	0.6102	0.5707	0.067*	
C36	0.2217 (4)	0.4827 (2)	0.5371 (2)	0.0459 (6)	
Cl36	0.14997 (12)	0.37906 (7)	0.65309 (6)	0.0650 (2)	

### Atomic displacement parameters (Å^2^)

**Table:**

	*U*^11^	*U*^22^	*U*^33^	*U*^12^	*U*^13^	*U*^23^
N1	0.0581 (13)	0.0448 (12)	0.0374 (11)	0.0013 (10)	−0.0110 (9)	−0.0257 (9)
C2	0.0491 (14)	0.0430 (13)	0.0377 (12)	0.0000 (11)	−0.0068 (10)	−0.0222 (11)
O2	0.0766 (14)	0.0497 (11)	0.0483 (11)	0.0148 (10)	−0.0283 (10)	−0.0281 (9)
C3	0.0419 (12)	0.0388 (12)	0.0302 (11)	−0.0033 (10)	−0.0057 (9)	−0.0157 (10)
C4	0.0504 (14)	0.0393 (13)	0.0345 (12)	−0.0049 (10)	−0.0047 (10)	−0.0177 (10)
C5	0.0551 (15)	0.0406 (13)	0.0372 (13)	−0.0008 (11)	−0.0103 (11)	−0.0113 (11)
Cl5	0.1182 (8)	0.0484 (4)	0.0584 (5)	0.0115 (4)	−0.0432 (5)	−0.0139 (4)
C6	0.0665 (18)	0.0371 (13)	0.0557 (16)	0.0052 (12)	−0.0096 (14)	−0.0228 (12)
C7	0.0644 (17)	0.0419 (14)	0.0500 (15)	0.0009 (12)	−0.0068 (13)	−0.0284 (12)
C8	0.0436 (13)	0.0435 (13)	0.0361 (12)	−0.0038 (10)	−0.0026 (10)	−0.0212 (11)
C9	0.0399 (12)	0.0341 (11)	0.0340 (11)	−0.0038 (9)	−0.0019 (9)	−0.0166 (9)
C10	0.0469 (13)	0.0360 (12)	0.0351 (12)	0.0021 (10)	−0.0104 (10)	−0.0156 (10)
C11	0.0456 (13)	0.0287 (11)	0.0329 (11)	−0.0020 (9)	−0.0061 (9)	−0.0133 (9)
C12	0.0447 (13)	0.0397 (12)	0.0381 (12)	−0.0058 (10)	−0.0012 (10)	−0.0182 (10)
Cl12	0.0485 (4)	0.0678 (5)	0.0578 (4)	−0.0124 (3)	0.0077 (3)	−0.0361 (4)
C13	0.0503 (15)	0.0683 (19)	0.0521 (16)	−0.0161 (14)	−0.0043 (13)	−0.0325 (15)
C14	0.071 (2)	0.082 (2)	0.0520 (17)	−0.0184 (17)	−0.0036 (14)	−0.0451 (17)
C15	0.0613 (18)	0.0677 (19)	0.0483 (15)	−0.0050 (14)	0.0028 (13)	−0.0364 (15)
C16	0.0408 (13)	0.0451 (14)	0.0415 (13)	−0.0020 (10)	−0.0053 (10)	−0.0193 (11)
Cl16	0.0433 (4)	0.1032 (7)	0.0631 (5)	0.0028 (4)	−0.0077 (3)	−0.0411 (5)
N21	0.0630 (14)	0.0297 (10)	0.0405 (11)	−0.0003 (9)	−0.0007 (10)	−0.0185 (9)
C22	0.0616 (16)	0.0313 (12)	0.0376 (13)	−0.0003 (11)	0.0006 (11)	−0.0164 (10)
O22	0.0968 (16)	0.0346 (9)	0.0476 (11)	0.0152 (10)	−0.0217 (11)	−0.0203 (9)
C23	0.0538 (14)	0.0291 (11)	0.0378 (12)	0.0022 (10)	−0.0015 (11)	−0.0153 (10)
C24	0.0586 (16)	0.0361 (13)	0.0421 (13)	0.0010 (11)	−0.0035 (12)	−0.0192 (11)
C25	0.0617 (17)	0.0485 (15)	0.0395 (14)	0.0050 (13)	−0.0093 (12)	−0.0160 (12)
Cl25	0.1096 (8)	0.0673 (5)	0.0607 (5)	0.0255 (5)	−0.0378 (5)	−0.0257 (4)
C26	0.0694 (19)	0.0619 (18)	0.0438 (15)	−0.0053 (15)	−0.0094 (13)	−0.0290 (14)
C27	0.0702 (19)	0.0472 (15)	0.0478 (15)	−0.0081 (13)	−0.0006 (13)	−0.0303 (13)
C28	0.0498 (14)	0.0355 (12)	0.0387 (12)	−0.0042 (10)	0.0040 (10)	−0.0185 (10)
C29	0.0515 (14)	0.0366 (12)	0.0337 (12)	−0.0051 (10)	−0.0003 (10)	−0.0178 (10)
C30	0.0601 (16)	0.0306 (12)	0.0365 (12)	0.0034 (10)	−0.0111 (11)	−0.0137 (10)
C31	0.0559 (15)	0.0325 (12)	0.0390 (12)	0.0039 (10)	−0.0152 (11)	−0.0187 (10)
C32	0.0595 (16)	0.0380 (13)	0.0397 (13)	0.0004 (11)	−0.0133 (11)	−0.0184 (11)
Cl32	0.0888 (6)	0.0524 (4)	0.0433 (4)	−0.0095 (4)	0.0018 (3)	−0.0231 (3)
C33	0.0693 (18)	0.0329 (13)	0.0535 (16)	−0.0012 (12)	−0.0223 (14)	−0.0148 (12)
C34	0.0660 (19)	0.0317 (13)	0.077 (2)	0.0092 (12)	−0.0240 (16)	−0.0275 (14)
C35	0.0593 (17)	0.0475 (15)	0.071 (2)	0.0038 (13)	−0.0068 (15)	−0.0374 (15)
C36	0.0572 (16)	0.0380 (13)	0.0453 (14)	−0.0020 (11)	−0.0082 (12)	−0.0210 (11)
Cl36	0.0868 (6)	0.0549 (4)	0.0561 (4)	−0.0154 (4)	0.0106 (4)	−0.0283 (4)

### Geometric parameters (Å, °)

**Table:**

N1—C2	1.360 (3)	N21—C22	1.352 (3)
N1—C8	1.398 (3)	N21—C28	1.397 (3)
N1—H1	0.8600	N21—H21	0.8600
C2—O2	1.219 (3)	C22—O22	1.220 (3)
C2—C3	1.508 (3)	C22—C23	1.509 (3)
C3—C10	1.329 (3)	C23—C30	1.331 (3)
C3—C9	1.457 (3)	C23—C29	1.456 (4)
C4—C5	1.378 (4)	C24—C25	1.378 (4)
C4—C9	1.383 (3)	C24—C29	1.382 (4)
C4—H4	0.9300	C24—H24	0.9300
C5—C6	1.387 (4)	C25—C26	1.383 (4)
C5—Cl5	1.736 (3)	C25—Cl25	1.737 (3)
C6—C7	1.373 (4)	C26—C27	1.385 (4)
C6—H6	0.9300	C26—H26	0.9300
C7—C8	1.375 (4)	C27—C28	1.372 (4)
C7—H7	0.9300	C27—H27	0.9300
C8—C9	1.397 (3)	C28—C29	1.404 (3)
C10—C11	1.475 (3)	C30—C31	1.467 (3)
C10—H10	0.9300	C30—H30	0.9300
C11—C12	1.391 (4)	C31—C36	1.388 (4)
C11—C16	1.395 (3)	C31—C32	1.398 (4)
C12—C13	1.378 (4)	C32—C33	1.374 (4)
C12—Cl12	1.732 (3)	C32—Cl32	1.733 (3)
C13—C14	1.370 (4)	C33—C34	1.370 (4)
C13—H13	0.9300	C33—H33	0.9300
C14—C15	1.369 (5)	C34—C35	1.381 (5)
C14—H14	0.9300	C34—H34	0.9300
C15—C16	1.373 (4)	C35—C36	1.382 (4)
C15—H15	0.9300	C35—H35	0.9300
C16—Cl16	1.734 (3)	C36—Cl36	1.733 (3)
			
C2—N1—C8	111.2 (2)	C22—N21—C28	111.3 (2)
C2—N1—H1	124.4	C22—N21—H21	124.4
C8—N1—H1	124.4	C28—N21—H21	124.4
O2—C2—N1	125.9 (2)	O22—C22—N21	126.6 (2)
O2—C2—C3	127.6 (2)	O22—C22—C23	126.5 (2)
N1—C2—C3	106.4 (2)	N21—C22—C23	106.9 (2)
C10—C3—C9	134.2 (2)	C30—C23—C29	134.6 (2)
C10—C3—C2	120.2 (2)	C30—C23—C22	120.1 (2)
C9—C3—C2	105.53 (19)	C29—C23—C22	105.2 (2)
C5—C4—C9	118.1 (2)	C25—C24—C29	118.2 (2)
C5—C4—H4	120.9	C25—C24—H24	120.9
C9—C4—H4	120.9	C29—C24—H24	120.9
C4—C5—C6	121.8 (2)	C24—C25—C26	122.0 (3)
C4—C5—Cl5	118.7 (2)	C24—C25—Cl25	118.4 (2)
C6—C5—Cl5	119.6 (2)	C26—C25—Cl25	119.6 (2)
C7—C6—C5	120.3 (2)	C25—C26—C27	120.0 (3)
C7—C6—H6	119.8	C25—C26—H26	120.0
C5—C6—H6	119.8	C27—C26—H26	120.0
C6—C7—C8	118.4 (2)	C28—C27—C26	118.5 (2)
C6—C7—H7	120.8	C28—C27—H27	120.8
C8—C7—H7	120.8	C26—C27—H27	120.8
C7—C8—C9	121.6 (2)	C27—C28—N21	129.1 (2)
C7—C8—N1	128.7 (2)	C27—C28—C29	121.5 (2)
C9—C8—N1	109.7 (2)	N21—C28—C29	109.5 (2)
C4—C9—C8	119.8 (2)	C24—C29—C28	119.8 (2)
C4—C9—C3	133.0 (2)	C24—C29—C23	133.0 (2)
C8—C9—C3	107.1 (2)	C28—C29—C23	107.2 (2)
C3—C10—C11	129.5 (2)	C23—C30—C31	128.8 (2)
C3—C10—H10	115.3	C23—C30—H30	115.6
C11—C10—H10	115.3	C31—C30—H30	115.6
C12—C11—C16	115.2 (2)	C36—C31—C32	115.6 (2)
C12—C11—C10	124.4 (2)	C36—C31—C30	120.8 (2)
C16—C11—C10	120.0 (2)	C32—C31—C30	123.4 (2)
C13—C12—C11	122.5 (2)	C33—C32—C31	122.4 (3)
C13—C12—Cl12	117.7 (2)	C33—C32—Cl32	116.8 (2)
C11—C12—Cl12	119.70 (19)	C31—C32—Cl32	120.63 (19)
C14—C13—C12	119.7 (3)	C34—C33—C32	119.7 (3)
C14—C13—H13	120.1	C34—C33—H33	120.1
C12—C13—H13	120.1	C32—C33—H33	120.1
C15—C14—C13	120.3 (3)	C33—C34—C35	120.4 (2)
C15—C14—H14	119.9	C33—C34—H34	119.8
C13—C14—H14	119.9	C35—C34—H34	119.8
C14—C15—C16	119.0 (3)	C34—C35—C36	118.7 (3)
C14—C15—H15	120.5	C34—C35—H35	120.7
C16—C15—H15	120.5	C36—C35—H35	120.7
C15—C16—C11	123.3 (2)	C35—C36—C31	123.2 (3)
C15—C16—Cl16	118.6 (2)	C35—C36—Cl36	117.9 (2)
C11—C16—Cl16	118.14 (19)	C31—C36—Cl36	118.92 (19)

### Hydrogen-bond geometry (Å, °)

**Table:**

*D*—H···*A*	*D*—H	H···*A*	*D*···*A*	*D*—H···*A*
N1—H1···O2^i^	0.86	2.09	2.919 (3)	162
N21—H21···O22^ii^	0.86	2.02	2.838 (3)	159

Symmetry codes: (i) −*x*+2, −*y*, −*z*+1; (ii) −*x*+1, −*y*, −*z*+1.
